# Exploring Large-Scale Patterns of Genetic Variation in the COI Gene among Insecta: Implications for DNA Barcoding and Threshold-Based Species Delimitation Studies

**DOI:** 10.3390/insects13050425

**Published:** 2022-04-30

**Authors:** Haiguang Zhang, Wenjun Bu

**Affiliations:** 1 College of Life Sciences, Linyi University, Linyi 276000, China; haiguangzhang123@163.com; 2 Institute of Entomology, College of Life Sciences, Nankai University, Tianjin 300071, China

**Keywords:** COI, DNA barcoding, Insecta, species delimitation

## Abstract

**Simple Summary:**

Insecta is the most diverse group in the kingdom Animalia, and it accounts for approximately two-thirds of all animals. The most commonly used gene for species delimitation in animals is cytochrome c oxidase subunit 1 (COI). We calculated the genetic distance of 64,414 insect species, downloaded from BOLD, and found that approximately one-quarter of the species of Insecta showed high intraspecific genetic variation (>3%). Owing to the high intraspecific genetic variation in insects, false positives may easily occur in threshold-based species delimitation based on the COI gene. Compared to the fixed thresholds, the thresholds that were calculated from the “threshOpt” and “localMinima” algorithms that are embedded in the Spider package are recommended in threshold-based species delimitation studies.

**Abstract:**

The genetic variation in the COI gene has had a great effect on the final results of species delimitation studies. However, little research has comprehensively investigated the genetic divergence in COI among Insecta. The fast-growing COI data in BOLD provide an opportunity for the comprehensive appraisal of the genetic variation in COI among Insecta. We calculated the K2P distance of 64,414 insect species downloaded from BOLD. The match ratios of the clustering analysis, based on different thresholds, were also compared among 4288 genera (35,068 species). The results indicate that approximately one-quarter of the species of Insecta showed high intraspecific genetic variation (>3%), and a conservative estimate of this proportion ranges from 12.05% to 22.58%. The application of empirical thresholds (e.g., 2% and 3%) in the clustering analysis may result in the overestimation of the species diversity. If the minimum interspecific genetic distance of the congeneric species is greater than or equal to 2%, it is possible to avoid overestimating the species diversity on the basis of the empirical thresholds. In comparison to the fixed thresholds, the “threshOpt” and “localMinima” algorithms are recommended for the provision of a reference threshold for threshold-based species delimitation studies.

## 1. Introduction

Insecta is the most diverse group in the kingdom Animalia, and it accounts for approximately 66% of all animals [1]. The estimated number of insect species is about 5.5 million (range: 2.6–7.8 million), of which only 1 million insect species are named, and 80% of the species have yet to be discovered [2,3]. Among Insecta, four orders (i.e., Coleoptera, Diptera, Hymenoptera, and Lepidoptera) account for 81% of all the described species of living insects [4]. Furthermore, insects have a long history on earth, and the origin of insects could date back to the early Ordovician (~479 million years ago) [5]. Although more and more studies have used multiple genes for species delimitation, single-locus data continue to dominate the DNA-taxonomy-related literature [6,7]. The most commonly used gene for species delimitation in animals is cytochrome c oxidase subunit 1 (COI) [8]. However, little research has comprehensively investigated the genetic divergence in COI among Insecta. Early studies indicate that the intraspecific genetic distances in mitochondrial genes were rarely greater than 2%, and most were less than 1% [9]. This conclusion was derived from phylogeographic analyses on a limited number of species. Hebert et al. (2003a) found that the distance between conspecific individuals of lepidopterans were always small, with an average intraspecific genetic distance of 0.25% [10]. Later, the intraspecific genetic variation in Insecta was mainly deduced from the DNA barcoding studies of various taxa [11,12,13,14,15,16,17,18,19,20,21,22,23,24]. To pursue the degree of species coverage, very few individuals (e.g., no more than three) were chosen for each species in the early DNA barcoding studies. Furthermore, the intraspecific genetic distance was significantly correlated with the geographical scale of the sampling [25]. Zhang et al. (2017) indicate that high intraspecific genetic distances (e.g., >3%) in COI were common when given comprehensive sampling (48.44% of 384 species), while this conclusion required further verification by more cases of insects [26].

The commonly used empirical thresholds for the threshold-based species delimitation of the COI gene include 1%, 2%, 2.2%, and 3% [7,10,24,27,28,29]. Furthermore, a high genetic distance of the COI gene is usually used to predict cryptic or new species [30,31,32,33,34,35,36,37,38,39,40,41]. It is widely accepted that the genetic variation in the COI gene has had a great effect on the final results of species delimitation studies. Meanwhile, the accuracy of the threshold-based approach critically depends on the level of overlap between the intra- and interspecific variations [42]. In many studies, unjustified and arbitrary threshold values (e.g., 2% and 3%) are frequently adopted from previous literature [43]. If the fixed threshold is relatively low, one species would be divided into two or more molecular operational taxonomic units (MOTUs). By contrast, a relatively high threshold may lead to the merging of two or more species into one species. Therefore, an optimal threshold that corresponds to the specific taxa is preferred, rather than a fixed empirical threshold.

In recent years, the COI data of Insecta in GenBank have been increasing rapidly with the emergence of DNA barcoding and the development of high-throughput sequencing techniques. The number of COI records deposited in GenBank has increased by a geometric average of 51% per year, from 8137 records in 2003, to nearly 2.5 million records by the end of 2017 [44]. Specifically, the number of Insecta COI data in GenBank was 5973 before January 2003; however, this number increased to 2,501,431 as of January 2022. Some researchers have been dedicated to analyzing the COI data. For instance, Hebert et al. (2003b) analyzed the genetic diversity in the COI across 2238 animal species [27]. Virgilio et al. (2010) compared the performance of DNA barcoding across six insect orders by using 15,948 COI sequences of 1995 species [45]. Bianchi and Gonçalves (2021) explored the genetic variation in the COI genes of 1068 species from Pentatomomorpha [46]. Nevertheless, the numbers of insect species and the taxon coverage that are reported in previous studies are still limited, which makes the evaluation of the genetic variation in COI among Insecta inaccurate. As a matter of fact, the Barcode of Life Data System (BOLD) owns more high-quality COI barcode data than the GenBank. Herein, the selection of COI data of Insecta from BOLD allowed us to comprehensively evaluate the genetic variation in COI among Insecta, which may provide new insights into COI-based DNA barcoding and threshold-based species delimitation studies.

## 2. Materials and Methods

### 2.1. Data Filtering

The Insecta COI data were downloaded from BOLD before 23 January 2021 by searching with the keyword “Insecta”. The raw data included 5,413,265 sequences. These sequences were first filtered by the following criteria: (i) sequences that had not been identified to the species level were deleted; (ii) sequences with names that included the keywords, such as “aff”, “cf”, “nr”, and “spp”, were eliminated; (iii) sequences without the label “COI-5P” were deleted; (iv) species with numbers of sequences less than three were excluded. Furthermore, the sequences were separated into different files according to the species names. The files that were grouped by species names were aligned by using MUSCLE [47]. As for the species that contained more than 100 sequences, 100 sequences were randomly selected and used for the subsequent sequence alignment. The files with insertions and deletions after the alignment (greater than or equal to one gap in the sequence alignment) were selected first, and then the sequences that lead to insertions or deletions were deleted. Furthermore, we translated the DNA sequences to amino acid sequences after the sequence alignment. The sequence files, including the stop codons, were subsequently deleted. We then calculated the K2P distances of the retained species by using the “dist.dna” function of the APE package [48]. The species that included an “NA” value in the genetic distance were also discarded. Finally, we deleted species with a maximum intraspecific genetic distance greater than the 95th-quantile percentile value.

### 2.2. Analysis at the Species Level

Following the data filtering, COI sequence data for 64,414 species files were obtained for the statistical analysis of the intraspecific genetic distance of the COI gene. The taxonomic category of each species was extracted from the BOLD system. Then, we grouped the 64,414 species to the corresponding orders. Orders with species numbers less than 1000 were pooled as “Others”. The intraspecific genetic distance among different orders was subsequently analyzed. We calculated the frequency distribution of the maximum intraspecific genetic distances for each order with a class interval of 0.01. The clustering analysis was performed with the refined “tclust” function (with “apply” changed to “alply” in the source code) of the Spider package [49] by using the thresholds of 1%, 2%, 2.2%, and 3%, respectively.

### 2.3. Analysis at the Genus Level

We first grouped the sequences according to the genus name, and we then selected those genera with species numbers greater than or equal to three. At the same time, we deleted the congeneric species with interspecific genetic distances of zero. These sequences were also aligned by using MUSCLE, and the files with insertions and deletions after alignment were filtered according to the abovementioned method. Afterward, we removed genera with less than three species. Furthermore, we deleted families that included only a single genus. Finally, 4288 genera (35,068 species) were obtained for the following analyses. We conducted the clustering analysis by using the refined “tclust” function, with fixed thresholds of 1%, 2%, 2.2%, and 3%, respectively. For comparison purposes, we also conducted the clustering analysis by using three kinds of flexible thresholds: the possible thresholds from the distance matrix (“localMinima” thresholds), the minimum congeneric interspecific genetic distances (“Mininter” thresholds), and the optimal thresholds (“Opt” thresholds). The “localMinima” thresholds were calculated by using the “localMinima” function of the Spider package. This function is based on the concept of the barcoding gap, where a dip in the density of the genetic distances indicates a transition between the intra- and interspecific distances. This method does not require prior knowledge of the species identity. The first local minimum was chosen for the subsequent calculations. The “Mininter” thresholds were calculated from the minimum congeneric interspecific genetic distances. Owing to the rounding-off of the minimum congeneric interspecific genetic distances in R, the “Mininter” thresholds were corrected by deducting a value of “0.00000001”. The “Opt” threshold was calculated by using the “threshOpt” function of the Spider package. When running over a range of thresholds (0.001–0.2; step length = 0.001), this function allows for the optimization of the threshold values by the minimization of the identification error rates. If the same minimum identification error rate was obtained from multiple thresholds, the mean value was selected.

Moreover, in order to compare the species delimitation results that were obtained from different methods, we calculated the match ratio by using the following formula: 2 ∗ Nmatch/(Ndelimited + Nmorph) [50,51], where Nmatch is the number of delimited species exactly matching the taxonomic species, Ndelimited is the number of delimited species using a particular method, and Nmorph is the number of morphologically defined species. As for each genus, the correspondence between species and OTUs can be divided into four categories: MATCH, SPLIT, MERGE, and MIXTURE, as described by Ratnasingham and Hebert (2013). All of the statistical analyses were performed in R v4.0.3 (R Core Team 2020) [52]. The graphics were drawn with the R package ‘ggplot2’ [53].

## 3. Results

### 3.1. Analysis Results at the Species Level

Among the 64,414 species, the maximum intraspecific genetic distances of 28,619 species (44.43%) ranged from 0 to 1%. By contrast, the numbers of species whose maximum intraspecific genetic distances exceeded 2% and 3% were 23,920 (37.13%) and 17,146 (26.62%), respectively. Approximately one-quarter of the species of Insecta showed high intraspecific genetic variation (>3%).

**Figure 1:**
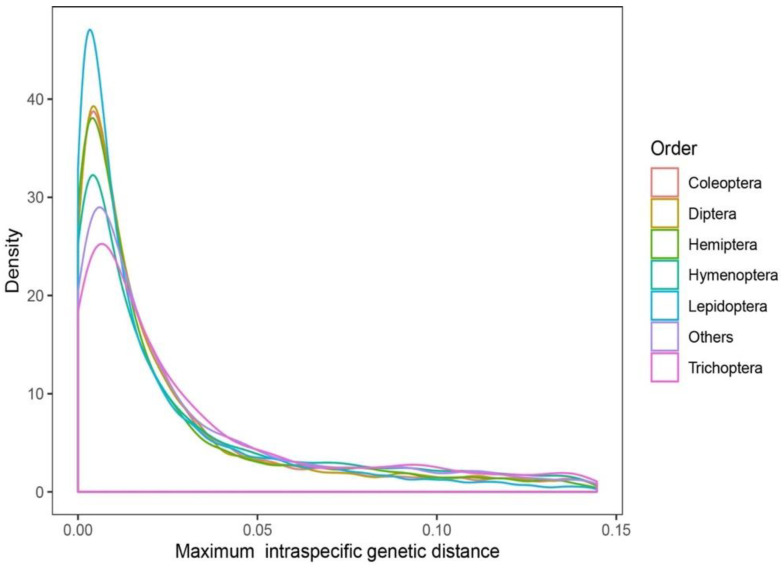
Frequency density of maximal intraspecific genetic distances of different orders.

For the orders that included more than 1000 species, the frequency densities of the maximum intraspecific genetic distances are shown in Figure 1. The percentages of the maximum intraspecific genetic distances over 3% for Coleoptera (8968 species), Diptera (9149 species), Hemiptera (3757 species), Hymenoptera (7977 species), Lepidoptera (29,529 species), and Trichoptera (1681 species) were 26.1%, 26.1%, 25.9%, 32.5%, 24.3%, and 34.6%, respectively. As for the orders whose species numbers exceed 100, the percentages of the maximum intraspecific genetic distances over 3% for Blattodea (250 species), Ephemeroptera (405 species), Neuroptera (212 species), Odonata (701 species), Orthoptera (829 species), Plecoptera (431 species), Psocodea (129 species), and Thysanoptera (130 species) were 36%, 35.3%, 23.1%, 25.7%, 35.3%, 42.2%, 24%, and 29.2%, respectively.

If the thresholds of 1%, 2%, 2.2%, and 3% were used in the clustering analysis, the 64,414 species could be divided into 113,055, 90,408, 87,875, and 81,864 clusters, respectively. Among them, 26,943 (23.83%), 17,483 (19.34%), 16,178 (18.41%), and 12,854 (15.70%) species can be divided into two or more clusters.

### 3.2. Analysis Results at the Genus Level

**Figure 2:**
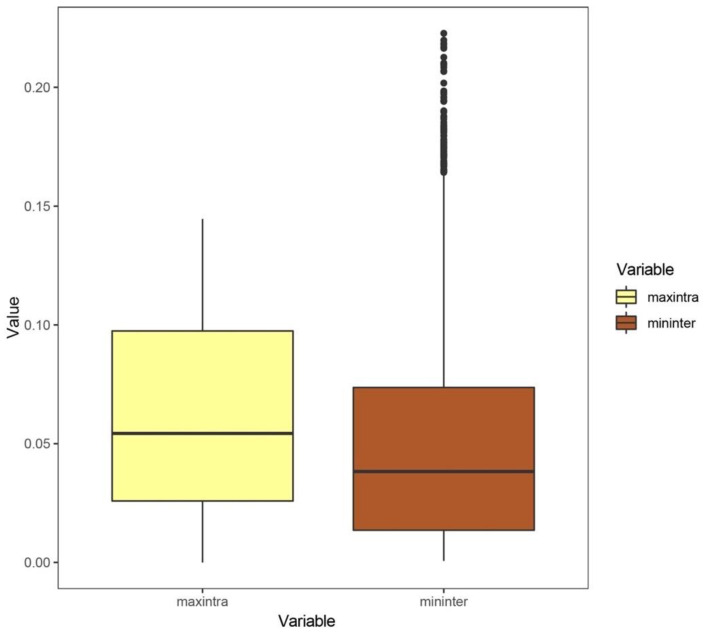
Intraspecific and interspecific genetic distances of 4288 genera. maxintra: the maximum intraspecific genetic distance of congeneric species; mininter: the minimum interspecific genetic distance of congeneric species.

We analyzed 4288 genera (35,068 species), and we found that the optimal thresholds for these genera ranged from 0.001 to 0.1590 (average value: 0.03416; median value: 0.02800). The detailed optimal threshold for each genus is shown in Table S1. The mean value of the maximum intraspecific genetic distances is larger than that of the minimum interspecific genetic distances of congeneric species. The boxplot of the maximum intraspecific genetic distances and the minimum interspecific genetic distances of congeneric species is shown in Figure 2.

If the thresholds of the clustering analysis were set to 1%, 2%, 2.2%, and 3% (empirical thresholds), the numbers of clusters were 57,950, 44,367, 42,603, and 37,698, respectively. Among the abovementioned thresholds, the number of clusters estimated with a threshold of 3% was the closest to that of morphologically defined species (overestimated ratio: 7.5%). Therefore, a threshold of 3% can be used for preliminarily estimating the insect diversity.

As for the 4288 genera (35,068 species), if the thresholds for the clustering analysis were set to 1%, 2%, 2.2%, and 3%, and the minimum interspecific genetic distances of congeneric species (0.068–22.27%) were applied, the 35,068 species could be divided into 57,950, 44,367, 42,603, 37,698, and 78,353 clusters, respectively. If the minimum interspecific genetic distances of congeneric species were set to greater than or equal to 1%, and the thresholds for the clustering analysis were set to 1%, 2%, 2.2%, and 3%, and the minimum interspecific genetic distances of congeneric species (1–22.27%) were applied, the 20,714 species could be divided into 35,243, 27,473, 26,541, 23,976, and 24,967 clusters, respectively. If the minimum interspecific genetic distances of congeneric species were set between 2% and 10% (interval: 1%), and the thresholds for the clustering analysis were set to 1%, 2%, 2.2%, and 3%, along with the minimum interspecific genetic distances of congeneric species, the numbers of clusters defined by the minimum interspecific genetic distances were smaller than those defined by the empirical thresholds (Table 1).

Therefore, if the minimum interspecific genetic distance of congeneric species was greater than or equal to 2%, it was possible to avoid overestimating the species diversity on the basis of the empirical thresholds.

If the empirical thresholds (1%, 2%, 2.2%, and 3%) were used for the OTU picking among the 4288 genera (35,068 species), the average match ratios were 0.4762, 0.6000, 0.6099, and 0.6182, respectively. By comparison, if the “localMinima” thresholds, the “Mininter” thresholds, and the “Opt” thresholds were applied in the clustering analysis, the average match ratios of them were 0.6342, 0.7135, and 0.7160, respectively.

**Figure 3:**
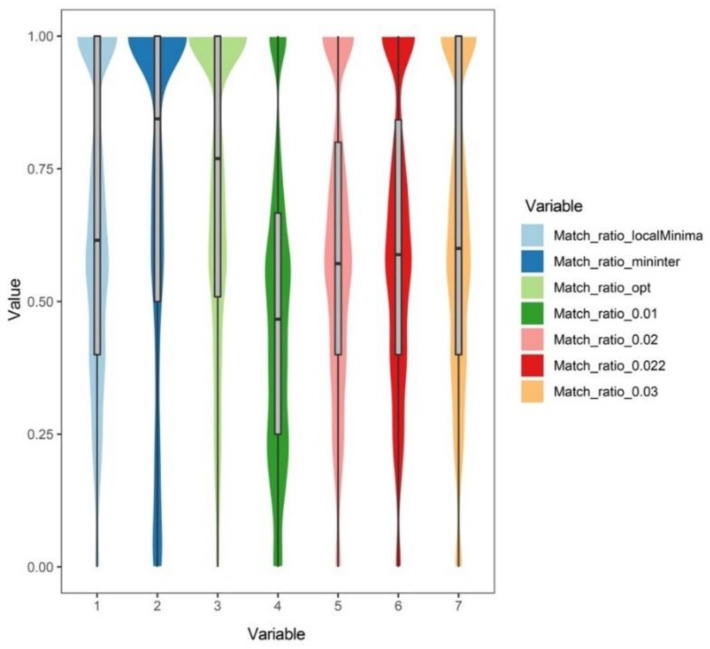
Match ratios of different methods. Match_ratio_*: the match ratio of 4288 genera in clustering analysis on the basis of the threshold of *. mininter: the minimum interspecific genetic distance of congeneric species; localMinima: the possible thresholds from the distance matrix; opt: the optimal thresholds.

The match ratios of 4288 genera based on different methods are shown in Figure 3.

**Figure 4:**
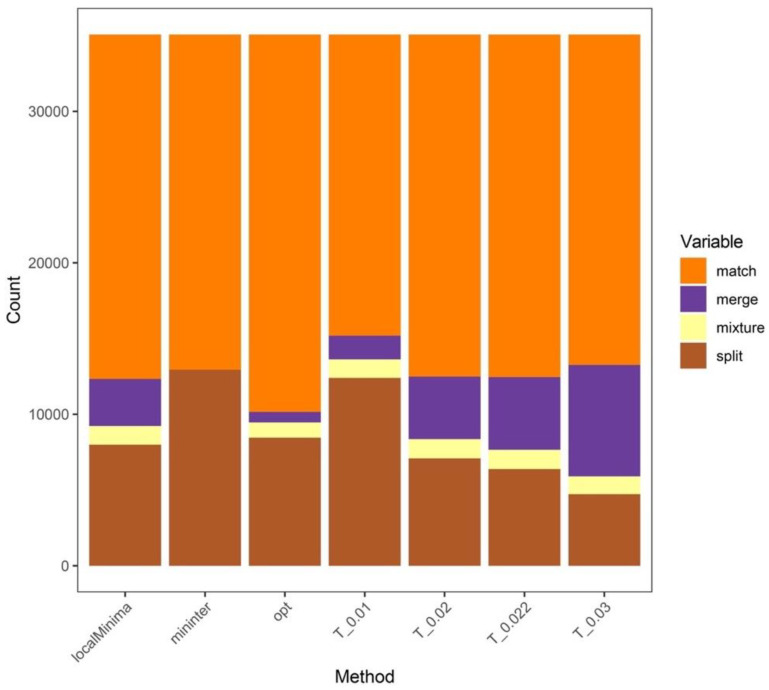
Numbers of “MATCH”, “MERGE”, “MIXTURE”, and “SPLIT” with different methods. T_*: the fixed thresholds of *. mininter: the minimum interspecific genetic distance of congeneric species; localMinima: the possible thresholds from the distance matrix; opt: the optimal thresholds.

In addition, the numbers of “MATCH” for 1%, 2%, 2.2%, 3%, the “localMinima” thresholds, the “Mininter” thresholds, and the “Opt” thresholds in the clustering analyses were 19,880, 22,580, 22,613, 21,811, 22,740, 22,125, and 24,912, respectively (Figure 4).

## 4. Discussion

Previous studies have shown that the intraspecific variation was significantly smaller than the interspecific genetic distance in COI among Insecta at the species level. However, our results indicate that the species with maximum intraspecific genetic distances above 3% account for about one-quarter of the 64,414 species. If we deleted the species with maximum intraspecific genetic distances over 5% or 10%, the species with maximum intraspecific genetic distances above 3% accounted for 12.05% or 22.58%, respectively. Herein, if empirical thresholds such as 2% and 3% are applied in the clustering analysis, the species diversity may be overestimated. Although the “dirty data” is inevitable, the inclusion of more species and sequences can reduce the impact of the probably mislabeled sequences to a certain extent. As time passes, the sequence number of the single species will also increase gradually, and more species with high genetic variation may be observed.

As for some widespread species with low dispersal rates and gene flows, extrinsic geographic isolation may lead to deeply intraspecific genetic differences [9]. Furthermore, insects have a long history, which can be traced back to more than 470 million years [5]. With the passage of time, the gradual accumulation of DNA mutations in the COI gene can lead to high genetic differentiation. In the meantime, the morphological characteristics of insects have not necessarily changed. Therefore, diverse groups of insects may show high intraspecific genetic variation.

Our results indicate that the high intraspecific variation may be common in insect species. Therefore, some defined cryptic species, on the basis of the high intraspecific variation in previous studies, may not be true. Meier et al. (2008) indicate that, if the aim was to predict cryptic species on the basis of genetic distance, the smallest interspecific distance had to be used [54]. The result indicates that, if the minimum interspecific genetic distance of congeneric species was greater than or equal to 2%, it was possible to avoid overestimating the species diversity on the basis of the empirical thresholds. Notably, although Srivathsan and Meier (2012) suggest that the calculation of the genetic distance should use the p-distance [55] rather than the K2P distance [56], Collins et al. (2012) indicate that the differences in the genetic distances between the best model and the K2P model were usually minimal, since the identification success rates were rarely affected by the model choice [57].

Because of the different evolutionary histories among diverged taxa, it is hard to find a fixed threshold that is suitable to all species. The inferences that are drawn from species delimitation studies should be conservative [58]. Notably, owing to the high intraspecific genetic variation in insects, false positives may easily occur in threshold-based species delimitation that is based on the COI gene. The proper threshold is essential when identifying specimens by using genetic-distance data [59]. The Spider package can calculate the optimized threshold (“threshOpt” algorithm), or it can generate a recommended one in the absence of taxonomic names (“localMinima” algorithm). By comparing the match ratios, the two methods also perform better than the clustering analysis on the basis of the fixed threshold. The selection of the threshold is important for the final results of species delimitation that is based on the clustering analysis in Insecta. Herein, the “threshOpt” and “localMinima” algorithms are recommended in the OTU picking in the COI-based species delimitation studies of insects. It is important to note that gene trees are not always equal to species trees. Mutanen et al. (2016) found a 12% incidence of non-monophyly in European Lepidoptera, while non-monophyletic species usually showed low interspecific genetic difference or high intraspecific variation [60]. The low match ratios of the clustering analysis may be relevant to the inherent limitations of the COI gene.

## 5. Conclusions

Approximately one-quarter of the species of Insecta showed high intraspecific genetic variation (>3%), and a conservative estimate of this value ranges from 12.05% to 22.58%. The selection of a threshold is essential for the final results of the clustering analysis. If empirical thresholds such as 2% and 3% are selected in the clustering analysis, the species diversity may be overestimated. Furthermore, if the minimum interspecific genetic distance of congeneric species was greater than or equal to 2%, it was possible to avoid overestimating the species diversity on the basis of the empirical thresholds. If a proper threshold was selected, the match ratio of the clustering analysis with a flexible threshold may outperform the fixed one. Herein, the “threshOpt” and “localMinima” algorithms can provide reference thresholds for threshold-based species delimitation studies.

## Figures and Tables

**Table 1:** The result of clustering analysis based on different thresholds.

Mininter	SN	M_0.01	M_0.02	M_0.022	M_0.03	M_mininter
≥0	35,068	57,950 (65%)	44,367 (27%)	42,603 (21%)	37,698 (7%)	78,353 (123%)
≥0.01	20,714	35,243 (70%)	27,473 (33%)	26,541 (28%)	23,976 (16%)	24,967 (21%)
≥0.02	16,037	27,653 (72%)	21,988 (37%)	21,298 (33%)	19,476 (21%)	18,171 (13%)
≥0.03	13,034	22,909 (76%)	18,139 (39%)	17,651 (35%)	16,474 (26%)	14,484 (11%)
≥0.04	10,454	18,584 (78%)	14,646 (40%)	14,237 (36%)	13,278 (27%)	11,366 (9%)
≥0.05	8395	15,029 (79%)	11,803 (41%)	11,466 (37%)	10,708 (28%)	8997 (7%)
≥0.06	6706	12,196 (82%)	9521 (42%)	9256 (38%)	8627 (29%)	7093 (6%)
≥0.07	5317	9810 (85%)	7633 (44%)	7406 (39%)	6908 (30%)	5569 (5%)
≥0.08	4177	7706 (84%)	6015 (44%)	5842 (40%)	5457 (31%)	4326 (4%)
≥0.09	3266	6093 (87%)	4724 (45%)	4589 (41%)	4273 (31%)	3365 (3%)
≥0.1	2395	4456 (86%)	3456 (44%)	3356 (40%)	3131 (31%)	2447 (2%)

Mininter: minimum interspecific genetic distances of congeneric species; SN: species number; M_*: the number of MOTUs based on the threshold of *. The values in parentheses represent overestimated ratios.

## Data Availability

The data presented in this study are openly available in FigShare at https://doi.org/10.6084/m9.figshare.19681632.v1 (accessed on 28 April 2022).

## Supplementary Materials

The following supporting information can be downloaded at: https://www.mdpi.com/article/10.3390/insects13050425/s1, Table S1: Detailed optimal thresholds for 4288 genera.
